# Genetic predictors of cultural values variation between societies

**DOI:** 10.1038/s41598-023-34845-x

**Published:** 2023-05-17

**Authors:** Justin Marcus, Ecesu Cetin

**Affiliations:** 1 College of Administrative Sciences and Economics, Koç University, Rumelifeneri Mah., Rumelifeneri Yolu, Sariyer, 34450 Istanbul, Turkey; 2 School of Medicine, Koç University, Istanbul, Turkey

**Keywords:** Human behaviour, Genetic association study

## Abstract

Associations between the STin2 and 5-HTTLPR polymorphisms within the serotonin transporter gene, SLC6A4, and culture across societies were examined. Based on an analysis of 75 primary studies (28,726 individuals), STin2 allelic frequencies were found to vary widely across countries, ranging from 26% in Germany to 85% in Singapore. Across 53 countries, and after controlling for all major environmental influences of culture, STin2 and 5-HTTLPR were found to explain 23.6% unique variance in monumentalism but none in individualism. Our findings evidence a significant role of genetics in predicting cross-societal cultural values variation, and potentially speak to the need for and importance of incorporating both nature and nurture in theories of cultural values variation across societies.

## Introduction

Perhaps resulting from a revolution in contemporary understanding of genetic variability, interest in research linking genes to social and psychological phenomena, termed “sociogenetics”, has burgeoned in the last decade^1^. Via this paradigm, evidence indicates that there are unique and meaningful cross-societal effects of genes on complex traits such as social stratification, social trust, intelligence test performance, educational attainment, voter turnout, happiness, and cognitive achievement^2–6^. Scholars have suggested that a deeper understanding of the interplay between genes and culture is essential for a holistic understanding of psychological processes^7^. Yet, very little is known about the role of genetics in cross-cultural differences, defined as differences in cultural values orientations between societies^1,8–10^. To the extent that prevailing theories of cultural values focus only on environmental factors ^e.g.,^^8–10^, a full accounting that considers the potential role of genetic differences between individuals from divergent populations is lacking. Indeed, the promise of finding genetic explanations for cultural values variation across societies is important. Insight into the genetic bases of said variation may help explain persistent differences in cultural values orientations between equally modernized and prosperous countries such as the US and Japan^8–10^. A holistic understanding involving both environmental and genetic influences of cross-cultural values variation between societies will enable scientists, students, and business and governmental decision makers to build more accurate templates for understanding social behavior and tackling social problems that arise resultant of differences between nations.

Consistent heritability estimates from twin and whole-genome studies suggest there is a reliable, albeit small, effect of genes on complex psychological traits such as personality and cultural values differences between individuals^11^. Comparing average population differences in genetic variability at the country or societal level has been advocated as an approach that helps address these small effects of genes at the individual level^5,12^. Accordingly, an emerging stream of research studying culture and genetics, via the paradigm of culture-gene co-evolution theory, has focused on associations between cultural values (e.g., individualism, tightness, power distance) and country-level variability in 5-HTTLPR^12–15^, a polymorphism in SLC6A4 (“Solute carrier family 6 member 4”), the gene containing information for production of the SERT protein that is responsible for transportation of serotonin from the synaptic cleft to its neuron^1^. However, results have been conflicting, possibly as a result of two important problems in this literature on the sociogenetics of culture.

First, a singular focus on only one polymorphism, 5-HTTLPR, has been a conceptual limitation restricting the accuracy of potential conclusions. The SLC6A4 gene is a complex system consisting of various polymorphic regions that each serve specific functions. Focusing on only one aspect of this system may lead to incorrect conclusions regarding associations between genes and culture. Crucially, there are *two* polymorphisms in SLC6A4 that play complementary roles in regulating serotonin receptivity, combining to either reduce or enhance serotonin uptake: 5-HTTLPR *and* STin2^16,17^. Second, extant studies lack model completeness and sample representativeness. Former studies on the sociogenetics of culture have only sporadically included environmental control variables, without consideration of the full range of environmental influences^18^. This is problematic because failing to account for environmental covariates leaves open the question whether documented associations between 5-HTTLPR and cultural values^12–15^ are robust. Additionally, said associations have also mostly not included populations from the “global South”, most notably sub-Saharan Africa.

Hence, and as detailed below, we address the just-noted issues by (1) synthesizing culture-gene co-evolution theory with advances in medical science involving allelic variation in serotonin receptivity to better clarify underlying associations between genes and cultural values; (2) conducting the first study on the effects of both polymorphisms in SLC6A4, 5-HTTLPR and STin2, on cultural values; (3) including *all* environmental factors that have been identified as important antecedents of cultural values; (4) including good representation of countries in the global South via reference to genetic maps of Eurasian, African, and Amerindian populations. We do so by examining population level associations between the SLC6A4 polymorphisms and two cultural values for which measurement-valid and scientifically rich data are available for most countries—individualism (the extent to which the individual is prioritized over the group) and monumentalism (the extent to which personality and behavior are viewed as interdependent and static as opposed to independent and malleable^19,20^).

In more detail, culture-gene coevolution theory suggests that environmental pressures giving rise to genetic adaptations also give rise to corresponding sociocultural adaptations^21^. Natural selection works to enhance the survival and reproductive fitness of individuals possessing genes linked to behavior patterns that yield success in a given social and physical environment^22^. Notably, genes affecting brain function are likely to influence the adoption and formation of cultural norms; conversely, culture may also shape the expression and selection of genes^21^. Consequently, cross-cultural variation between societies may be a function of genetic variation, with different patterns of gene-brain interactions giving rise to cultural differences between populations^7,23^. These genetic variation patterns between populations are also unlikely to be a result of chance (i.e., genetic drift) because similar patterns of gene-brain interactions have been found worldwide in ecologically threatening regions that are geographically isolated, involving SLC6A4^7,15^.

Two Variable Number of Tandem Repeat (VNTR) polymorphisms in SLC6A4, 5-HTTLPR and STin2, work together to increase SERT protein transportation from the SLC6A4 gene, combining to reduce or enhance serotonin uptake^17,24–26^. 5-HTTLPR has two allelic variants including the S (“short”) and L (“long”) alleles. Individuals with a homozygotic S-allele have lower serotonin receptivity than individuals with a homozygotic L-allele. The L-allele causes more efficient SLC6A4 transcription than the S-allele. SERT translation is decreased among homozygotic S-allele carriers, vice versa for homozygotic L-allele carriers^27–29^. STin2, responsible for SERT protein translation also^30^, is in intron 2 of the SLC6A4 gene and consists of 9, 10, or 12 repeat units, (STin2.9, STin2.10, and STin2.12). STin2.9 is quite rare, but almost everyone has either STin2.10 (henceforth, “10/10”) or STin2.12 (henceforth, “12/12”). Like the S-allele, 12/12 increases SLC6A4 transcription and decreases SERT translation; like the L-allele, 10/10 leads to more efficient SLC6A4 transcription and increased SERT translation^17,31^. Overall, both polymorphisms have been linked to similar outcomes including mental health problems, depression, suicide, and nicotine dependence^29,30,32–34^. Although underlying biological mechanisms for these findings are not fully understood yet^35,36^, because 12/12 and the S-allele both decrease SERT protein translation, it may generally be surmised that they should have similar effects on social outcomes and vice versa for 10/10 and the L-allele, which increase SERT translation^17,31^. Hence, potential associations between cultural values and STin2 may be informed by the extant research on 5-HTTLPR.

The S-allele is associated with enhancement of a wide range of cognitive functions, notably improved decision-making through better probabilistic and temporal discounting^37^. It promotes hypervigilance, leading to more anxiety, heightened responses to emotional stimuli, and more attention to negative information but also more environmental monitoring^37–39^. Consequently, individuals with homozygotic S-alleles are more risk-averse and more loss-averse than those with homozygotic L-alleles^40,41^. Thus, because national culture is closely related to aggregated personality traits^5^, it is reasonable to expect that societies typified by a greater prevalence of S-allele (or likewise, 12/12) carriers will also be more risk-averse, favoring cultural norms that embody more social harmony and social control^13^ (e.g., collectivism, monumentalism). Indeed, environmental threats that select for less serotonin uptake in the population also have been evidenced to covary with “survivalist” cultural tendencies dictating greater social cohesion such as the creation of strong hierarchies, tightly knit societies, and the prevalence of strong norms^14,15,42^. Therefore, we predict that SLC6A4 polymorphisms associated with reduced serotonin uptake (S-allele and 12/12) will be positively associated with monumentalism (more social control) but negatively associated with individualism (less social harmony); vice versa for 10/10, which increases serotonin uptake^16,17^. Going further, because culture-gene co-evolution theory maintains that associations between allelic and cultural variability are independent of environmental factors^21–23^, we predict that 5-HTTLPR and STin2 will explain unique variance in individualism and monumentalism beyond the effects of all essential environmental factors.

Summarily, we conduct the most comprehensive study to date on the confluence of cultural values and genetics, providing insight into specific patterns of genetically driven cross-cultural differences for a set of 28 countries representing 57% of the world population. First, we review the medical literature and aggregate data from 75 studies representing 28,726 individuals to determine the prevalence of the STin2 polymorphism across said countries. Ours is the first tabulation of STin2 across countries, providing a wealth of information on serotonin receptivity across populations that can be used by scholars of sociogenetics. Further, we use genetic mapping of the populations spanning the various continents to provide estimates of the STin2 polymorphism in another 27 countries that also have data available for S-allele prevalence. We thereby greatly expand the country-level database available for scholars to study SLC6A4, the serotonin transporter gene, while also ensuring that any estimates we provide are based on genetically comparable populations. Most importantly, because environmental factors exert powerful influences on cultural values and may in fact be responsible for both cultural *and* allelic variation^42–46^, we draw upon conceptual advancements in ecological psychology^18^ to statistically model 14 environmental factors including: geographical latitude, axial orientation, climatic demands, rainfall steadiness, historical/contemporary prevalence of disease, social diversity including ethnic/racial, linguistic, and religious diversity, population density, urbanization, wealth differences both between and within countries, and social conflict. This set subsumes *all* environmental covariates included in former studies of 5-HTTLPR^12–15^ and no other factors beyond these have been identified as important antecedents of cultural values^8^. Thus, we control for most confounds to statistically test the supposition that there is a genetic explanation for cross-cultural values variation between societies. To be clear, we do not make any inferences about a causal link between genes and culture among individuals. Rather, we model associations using a correlation and regression approach, based on overall population-wide data, focused only on *predicting statistical variation in cultural values orientations*.

## Results

### STin2 distributions across countries

**Figure 1:**
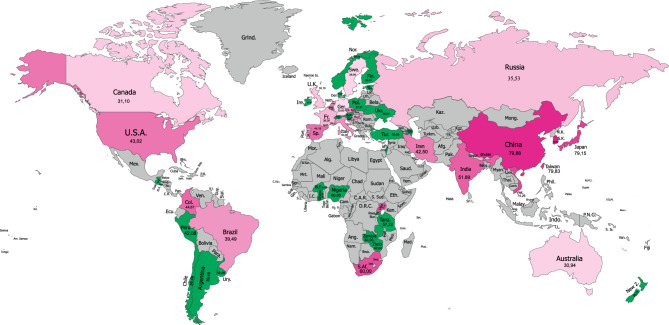
STin2.12 (12/12) allelic prevalence worldwide. Numbers shown represent estimated population percentages. Note: Countries with primary data available are shaded in red and countries with estimated data available are shaded in green. Map generated with CorelDRAW (Ver. 2019), available at https://www.coreldraw.com/en/.

Stin2 prevalence for all countries in our study are displayed in Fig. 1 (for 12/12) and 2 (for 10/10). A detailed tabulation of these countries and the primary studies they are based on is provided in Tables S1 and S2 (see "Supplementary Information"). 28 countries in our study had primary data on STin2—these are listed in the top half of Table S1. Another 27 countries were estimated based on genetic maps of the populations within these countries, via reference to the scientific literature delineating genetic overlaps between and within Eurasian, African, and Amerindian populations^47–51^ (see “Method” section for more details). Countries for which STin2 data were estimated are listed in the bottom half of Table S1.

**Figure 2:**
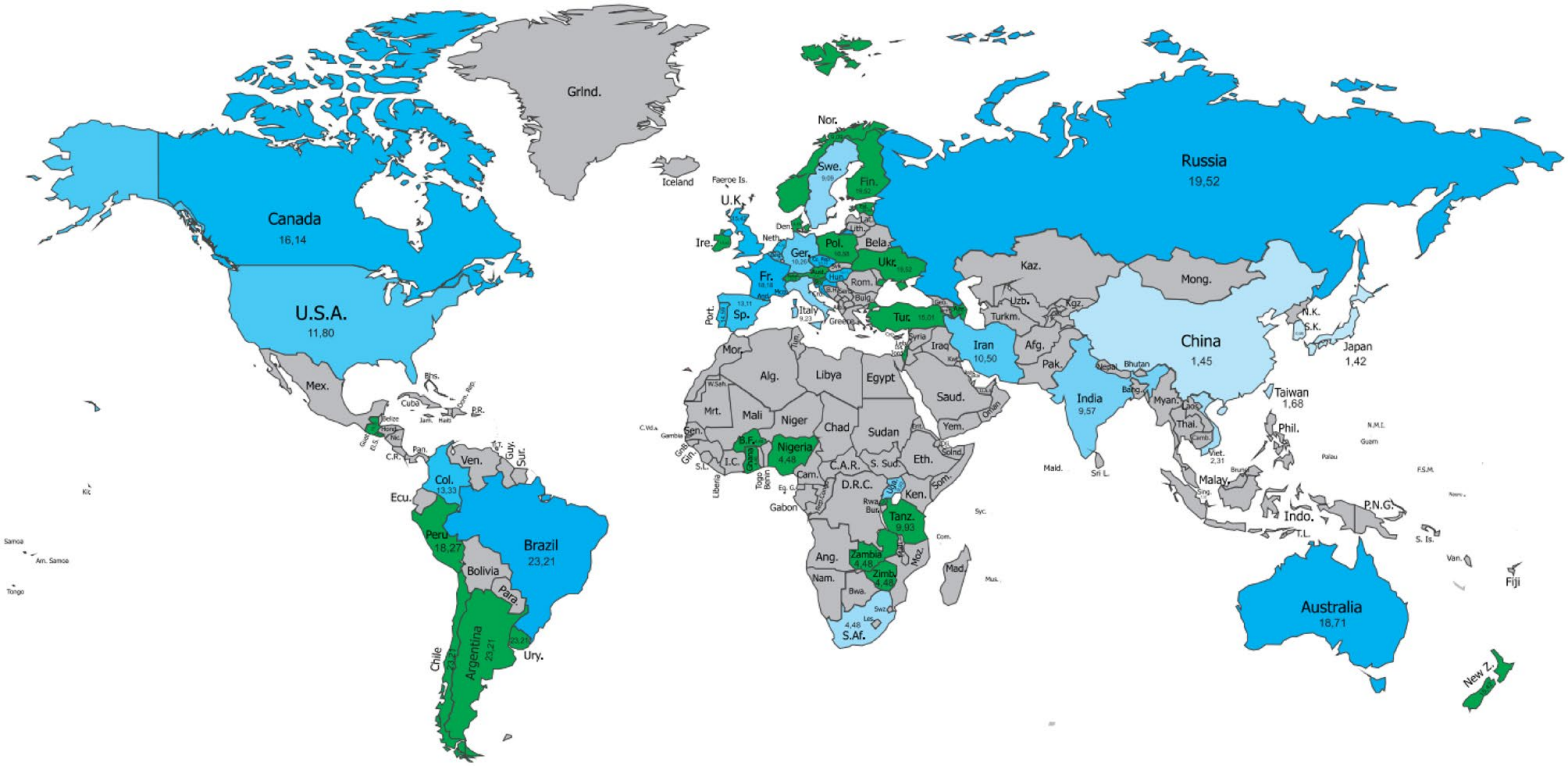
STin2.10 (10/10) allelic prevalence worldwide. Numbers shown represent estimated population percentages. Note: Countries with primary data available are shaded in blue and countries with estimated data available are shaded in green. Map generated with CorelDRAW (Ver. 2019), available at https://www.coreldraw.com/en/.

An examination of Figs. 1 and 2 reveals an interesting pattern. The five countries or regions with the highest prevalence of the 12/12 allele all have predominantly East Asian populations, including China (80%), Japan (79%), Singapore (85%), South Korea (84%), and Taiwan (80%). In contrast, the five countries with the lowest prevalence of the 12/12 allele all have mostly European populations including Australia (31%), Canada (31%), Germany (26%), Sweden (29%), and the UK (35%). Overall, Asian and African countries or regions have the highest prevalence of the 12/12 allele and European countries have the lowest. When considering 10/10 allelic prevalence, the five lowest countries/regions are East Asian, including China (1%), Japan (1%), South Korea (1%), and Taiwan (2%), and Southeast Asian (Vietnam; 2%); the five highest countries mostly also have predominantly European populations, including Australia (19%), Brazil (23%), Croatia (16%), France (18%), and Russia (20%). The 12/12 allele is much more prevalent in the population than the 10/10 allele.

### Correlation analyses

Correlation analyses were conducted in SPSS (Ver. 28). A detailed tabulation of the means, standard deviations, and intercorrelations between all study variables is provided in Table S3 (see "Supplementary Information"). As expected, the two STin2 alleles, 12/12 and 10/10, are very strongly and negatively related to each other (*r* = − 0.75, *p* < 0.001). As expected also, given that they are in different regions of SLC6A4, neither of the STin2 alleles are strongly correlated with S-allele prevalence (for 12/12: *r* = 0.26, *n. s.*; for 10/10: *r* = − 0.05, *n. s.*). To the extent that all correlations between the genetic variables are in logically expected directions and are consistent with prior research, we may have some confidence that the currently presented genetic data have measurement validity.

As shown in Table S3, directions of correlations between the SLC6A4 polymorphisms and the two cultural values orientations are generally in theoretically expected directions. S-allele and 12/12 allelic prevalence are positively correlated with monumentalism (a cultural value associated with more social control); this association is significant only for the S-allele (for 12/12: *r* = 0.07, *n. s.*; for S-allele: *r* = 0.58, *p* < 0.01). However, only 12/12 is, as expected, negatively correlated with individualism (a cultural value associated with less social harmony); S-allele prevalence is positively correlated with individualism, although this latter correlation is not statistically significant (for 12/12: *r* = − 0.52, *p* < 0.01; for S-allele: *r* = 0.22, *n. s.*). On the other hand, the 10/10 allele is, as expected, positively correlated with individualism and negatively correlated with monumentalism; the correlation is significant for individualism but not monumentalism (for individualism: *r* = 0.26, *p* = 0.05; for monumentalism: *r* = − 0.08, *n. s.*). Overall, findings provide some support for the notion that 12/12 and the S-allele are positively associated with cultural values that promote social harmony or social control (monumentalism) and negatively with those that do not (individualism), vice versa for 10/10.

### Regression analyses

Hierarchical regression analyses were conducted in SPSS (Ver. 28) to test the effects of the SLC6A4 polymorphisms on cultural values after accounting for the effects of environmental factors, with country as the unit of analysis. This analysis helps shed light on concerns surrounding the validity of previously evidenced correlations between SLC6A4 polymorphisms and cultural values orientations^12–15^. The regression analysis was conducted in three steps. All control variables were included in Step 1—this step indicates the amount of cultural values variance explained by factors other than the SLC6A4 polymorphisms and serves as a baseline to compare the unique contributions of the polymorphisms in explaining values variation between societies. The S-allele percentage was included in Step 2—this step indicates the unique contribution of the S-allele to values variation between societies after accounting for the control variables. Finally, 12/12 and 10/10 allelic percentages were included in Step 3—this step indicates the unique contribution of STin2 to cultural values variation between societies after accounting for the control variables and S-allele prevalence. Japan and New Zealand were excluded from the regression analyses because they lacked data on axial orientation. Findings are thus based on the remaining 53 countries.

**Figure 3:**
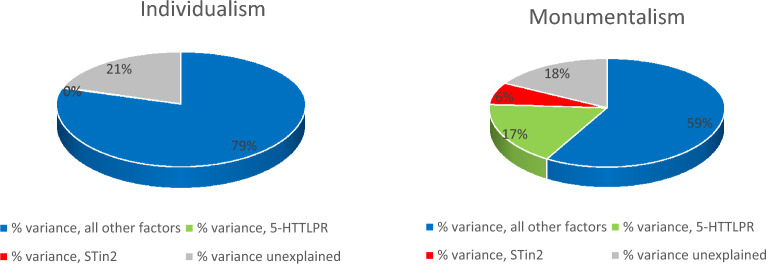
Percentage variance explained in cultural values by the SLC6A4 polymorphisms and other factors.

A summary snapshot displaying the main findings of interest is displayed in Fig. 3. Detailed results for the hierarchical regression analyses are provided in Table 1. The table lists the effects of the individual predictors (standardized regression coefficients) on individualism and monumentalism, the amount of variance explained in each step on individualism and monumentalism (*Adjusted R*^*2*^), the incremental variance explained by the SLC6A4 polymorphisms on individualism and monumentalism in each of Steps 2 and 3 (*Δ R*^*2*^), and a test statistic (*F*) indicating whether said variance explained for individualism or monumentalism is statistically significant. Statistically significant effects are bolded.

**Table 1:** Full hierarchical regression results.

Independent variables	Cultural Values Orientations	
	Individualism	Monumentalism
Step 1: Control variables^a^		
Latitude	− 0.012	0.275*
Axial orientation	− 0.006	0.036
Climatic demands	0.023	0.107
Rainfall steadiness	0.123	0.275*
Pathogen prevalence average	− 0.356*	− 0.030
Social diversity	− 0.079	0.114
Logged population density	− 0.160	− 0.138
Urbanization rate	0.131	0.106
GDP PPP	0.279*	0.174
Gini coefficient	− 0.136	− 0.366***
Number of territorial conflicts	0.008	0.112
* Adjusted R*^*2*^	0.792	0.585
* F*	19.032***	7.665***
Step 2: 5-HTTLPR		
% S-allele	0.104	0.536***
* Adjusted R*^*2*^	0.794	0.759
* ∆ R*^*2*^	0.002	0.174
* F*	1.356	30.538***
Step 3: STin2 added		
% 12/12	0.011	0.559***
% 10/10	0.038	0.191
* Adjusted R*^*2*^	0.784	0.821
* ∆ R*^*2*^	0.000	0.062
* F*	0.052	8.013***

^+^*p* < 0.10; * *p* < 0.05; ** *p* < 0.01; *** *p* < 0.001.

^a^Standardized regression coefficients for the control variables are from Step 3; *n* = 53 countries.

As shown in Table 1, the effects of pathogen prevalence and GDP PPP on individualism are statistically significant. The environmental control variables collectively explain 79.2% of the cultural values variation in individualism. None of the SLC6A4 polymorphisms (S-allele, 12/12, and 10/10) are significantly associated with individualism after controlling for the environmental factors in Step 1. These polymorphisms do not explain additional variation in individualism beyond the effects of the environmental factors, and collectively account for close to 0% of the variance in this cultural values dimension. Turning to monumentalism, the effects of latitude, rainfall steadiness, and the Gini coefficient are statistically significant. The environmental control variables collectively explain 58.5% of the cultural values variation in monumentalism. Effects of the S-allele and the 12/12 allele on monumentalism are statistically significant. The S-allele explains an additional 17.4% of the variation in monumentalism beyond the effects of all environmental factors, and the 12/12 allele explains an additional 6.2% of the variance beyond this. Collectively, the SLC6A4 polymorphisms explain 23.6% additional variation in monumentalism beyond the effects of the environmental factors, indicating a strong and potentially important cultural antecedent.

#### Robustness tests

There are several limitations with our statistical model. Four countries including France, Italy, Singapore, and Sweden, and one region, Taiwan, had only small sample sizes for STin2 frequency estimates in the population, raising the possibility that small sample sizes for population genetic estimates may have confounded study results. Four countries including Burkina Faso, El Salvador, Tanzania, and Zambia relied on estimated data on both genetic variables and cultural dimensions, thereby raising the possibility that data dependencies may have confounded study results. Twenty-seven countries (all countries listed in the bottom half of Table S1) did not include primary data on STin2, raising the possibility that study results may have been confounded by our use of genetic maps to “guess” STin2 estimates on genetically similar countries where no such primary data were in fact available. We conducted robustness tests to address all these potential limitations with our statistical model. Results are provided in Tables S4–S6 (see "Supplementary Information").

As shown in Table S4, study findings did not change after excluding countries with small sample sizes for STin2 frequency estimates in the population. In line with results in Table 1, neither S-allele, 12/12, nor 10/10 prevalence significantly predicted individualism after controlling for the environmental factors. As before, both S-allele and 12/12 prevalence significantly predicted monumentalism beyond the effects of environmental factors.

As shown in Table S5, study findings changed slightly after excluding countries with estimated data on both genetic variables and cultural dimensions. After controlling for environmental factors, the effect of S-allele prevalence on individualism was marginally significant; as before, neither 12/12 nor 10/10 were significant. As before too, both S-allele and 12/12 prevalence significantly predicted monumentalism beyond the effects of environmental factors.

As shown in Table S6, study findings became even stronger after excluding countries with estimated data on STin2. In line with results in Table 1, neither S-allele, 12/12, nor 10/10 prevalence significantly predicted individualism after controlling for the environmental factors. However, in addition to the S-allele and 12/12, 10/10 also had a statistically significant effect on monumentalism; taken together, the effect of the SLC6A4 polymorphisms on monumentalism dwarfed the effects of all environmental factors, accounting for almost half of the variation (48.9%) in this cultural value.

Overall, results of the robustness tests converge toward results reported in Table 1, ruling out the possibility that small sample sizes, data dependencies, or using genetic maps to estimate data for countries without primary data available for STin2 may have confounded the study results. These results demonstrate that there is a robust and practically meaningful genetic explanation for cultural values variance between societies as a function of naturally evolving differences in serotonin uptake across geographically distinct human populations.

#### Multicollinearity analyses

The small variable-observation ratio may have led to biased regression coefficients resulting from multicollinearity between study variables. Therefore, multicollinearity analyses were conducted to rule out this potential confound. Across all models (Table 1, Tables S4–S6), analyses indicated no multicollinearity between study variables, with all variance inflation factors below 10, ruling out the small variable-observation ratio as a study confound.

## Discussion

Synthesizing culture-gene co-evolution theory with advances in medical science involving allelic variation in serotonin receptivity, we conducted the most comprehensive study to date on cross-societal cultural values variation and genetics. We posited and found some support for the notion that there is a genetic explanation for a portion of the cultural values variation across societies. Notably, 12/12 and S-allele prevalence, both responsible for decreased serotonin uptake, were found to explain almost a quarter of the variation in monumentalism but no variance in individualism. These results held even after controlling for all key environmental factors and subjecting the data to stringent robustness tests.

The fact that the SLC6A4 polymorphisms did not predict individualism converges with previous research indicating that S-allele prevalence does not predict individualism-collectivism after other influences are accounted for^12,52^. Our results also replicate previous research indicating S-allele prevalence to be an important predictor of flexibility-monumentalism^12^. However, we go further and show that in addition to the S-allele, 12/12 is also perhaps an equally important predictor. Taken together, our findings suggest that the SLC6A4 polymorphisms may be very important influences of cultural values that concern social control, such as flexibility-monumentalism, but of no consequence for cultural values concerning social harmony such as individualism-collectivism. Indeed, the fact that former studies have found robust associations between S-allele prevalence and other cultural values related to social control such as social hierarchy and social constraint^14,15^ buttresses this notion. Future research is needed to deeper investigate potential associations between serotonin receptivity and different types of cultural values ^e.g.,^^8–10^.

Coupled with evidence that there are vast differences across countries on frequencies of the STin2 alleles, ranging from 26% in Germany to 85% in Singapore for 12/12, our findings speak to the potential importance of incorporating both nature and nurture in the study of culture. Given evidence associating values and genetic variation, theories on antecedents of cultural values ^e.g.,^^8,9^ may now be able to add another focal predictor: population allelic variation in SLC6A4, the serotonin transporter gene. Scholars studying cross-societal cultural values differences across countries may easily include this factor into their predictive models by referring to the STin2 data we provide; countries not represented in our data may be estimated using the genetic mapping method we have introduced.

A genetic component to cultural variability across societies helps to explain the persistent cross-cultural differences that have been found between equally modernized and wealthy countries such the US and Japan and suggests a potential roadblock toward global cultural convergence. Illustratively, cross-cultural scholars have found evidence for global convergence toward individualism and related values across the world’s countries, and speculate that at the present rate of change, cross-societal cultural values differences may altogether disappear by 2050^53^. Our findings, however, argue for the opposite position, indicating that the world’s societies will yet be quite divergent in their cultural values orientations given stubborn genetic differences between populations. Future research on the potential role of genetic variation in cultural values change across societies is needed.

Interestingly also, although the broad pattern suggests continental differences in the genetic divide, with East/Southeast Asia anchoring the high-end of 12/12 prevalence and Europe the low end, there are differences even within continents. Focusing on the continent with the most country-specific data available, Europe, there are large differences even between neighboring countries, with 12/12 allelic percentages ranging from 25% in Germany to 35% in neighboring France and 40% in the Netherlands and Belgium. Indeed, a 10–15% difference is quite substantial given how geographically close and historically intertwined these countries are; by way of comparison, the East Asian countries are all within ≈ 5% of each other. Could these genetic differences help explain persistent cultural values variation that has been found across Europe?^9,10^ Future research examining associations between cultural values variation and serotonin receptivity is needed to best answer this question.

### Limitations

Because we used population-wide metrics for environmental antecedents, polymorphisms, and cultural values, our study is strictly correlational only—a causal link between serotonin receptivity and values cannot be presently inferred. Ideally, individual-level data on the studied polymorphisms, coupled with population-wide metrics on environmental antecedents and cultural values, ought to be paired in a hierarchical linear model to make inferences regarding said causal link. We are unable to do this because the polymorphic data do not exist at the individual- but only at the sample-level (see Table S2). Hence, we maintain only that the SLC6A4 gene is an important factor in predicting cultural values *variance* (i.e., a statistical, not causal, explanation). On that note, we caution the reader to not derive a deterministic understanding of culture based upon a reading of our paper. We take the position that both nature and nurture are important antecedents of culture, and we hope that more research on this issue will spur forward.

Likewise, lacking individual-level data on specific polymorphisms, we cannot rule out the possibility that sociocultural differences between neighboring countries such as France and Germany may simply be resultant of differential ancestral origins, as opposed to genetic variation in serotonin receptivity. For instance, Bell Beaker culture was predominant in Germany but not so much in France^54^, leaving open the possibility that these countries evolved different cultural values simply because they were populated by people with different cultural practices to begin with. Future research disentangling the effects of ancestral origin and allelic variation in serotonin receptivity is needed to resolve this nature-nurture question.

Finally, we acknowledge that assigning a certain percentage in allelic variation for a given country is an imperfect method of estimating the actual allelic variation in countries with highly diverse populations such as Singapore, New Zealand, the UK, and the USA. Unfortunately, the primary studies from the medical science literature (see Table S2) by and large did not include detailed sample demographic breakdowns by race/ethnicity, precluding us from creating more refined estimates of cross-societal allelic variation in STin2. Nevertheless, the genetic maps we referenced^47–51^, particularly for Eurasian populations, suggest that many ethnic groups share common genetic origins (e.g., Finns, Russians, and Turks fall in the same genetic group^49^), thereby somewhat alleviating this concern. Moreover, it is unlikely that estimation sensitivity could rule out the effects we found considering the sheer size of said effects, whereby the SLC6A4 polymorphisms accounted for almost a quarter of the variation in monumentalism but close to zero in individualism (see Table 1). The robustness of our results hence lends some measure of confidence to our conclusions.

## Method

### STin2 literature review

PubMed was searched using the term “STin2” in February and March 2020; the search was then updated in March 2021. One hundred and fifty-three potential studies were identified for inclusion. To be included, studies must have been published in English and provided relevant sample statistics, including (a) the number of participants with STin2.12/STin2.12 (“12/12”), (b) the number of participants with STin2.10/STin2.10 (“10/10”), and (c) the total number of participants. Seventy-five studies met our inclusion criteria. The 75 studies are listed in Table S2 (see "Supplementary Information"). Information on country of origin, sample type, total sample size, and the total number of sample participants carrying both 12/12 and 10/10 STin2 alleles are also listed. As shown, primary STin2 data were available for 28 countries (28,726 individuals), representing parametric estimates for ≈ 57% of the world population.

### Calculation of country-specific percentages of the STin2 alleles

Across all countries, some samples were clinical (defined as samples whose members have specific ailments or comorbidities such as depression or cancer), whereas others included only healthy adults. To confirm genetic equivalencies between clinical and non-clinical samples, we compared the number of individuals with 12/12 and 10/10 alleles present in each type of sample. Independent-samples t-tests indicated no statistically significant differences between clinical and non-clinical samples on either the 12/12 (*t *_*(115)*_ = − 1.146, *p* = 0.254) or 10/10 alleles (*t *_*(115)*_ = 0.367, *p* = 0.715). These non-significant results are consistent with the extant medical literature indicating similar frequencies of the STin2 alleles between clinical and non-clinical samples^27,55^. Therefore, both clinical and non-clinical samples were summed to calculate STin2 percentages across specific countries.

For each country, the number of participants with 12/12 and/or 10/10 STin2 alleles was divided by the total number of sample participants to calculate a country-specific percentage. If a country had data from more than one study, all the participants were taken into consideration, with percentages calculated using the total participants carrying each allele, respectively, and divided by the total number of participants included in that country. For the United Kingdom, data were available separately for both England and Scotland. Given large discrepancies in population between these two parts of the UK, 12/12 and 10/10 percentages were multiplied by 0.90 for England, and 0.10 for Scotland, and then summed. The final calculated percentages for all 28 countries with primary data available are listed in the top half of Table S1 (see "Supplementary Information").

### STin2 percentage estimates via genetic mapping

Following prior research^12^, we estimated an additional 27 countries based on the available primary data. To ensure that all our estimates are valid, we utilized population genetic mapping of the continents to build genetically accurate estimates of STin2 allelic prevalence in each country. Because hypotheses involve comparing the relative effects of 5-HTTLPR and STin2, we limit estimates to 27 additional countries that also have information on S-allele prevalence^12^. STin2 (12/12 and 10/10) prevalence estimation procedures for these 27 countries are detailed below, by continent. Estimates are provided in the bottom half of Table S1 (see "Supplementary Information").

#### Africa

A genetic map of Africa is provided by Tishkoff et al.^47^. Using these data, estimates for Burkina Faso, Ghana, Nigeria, Zambia, and Zimbabwe are made based on South Africa; estimates for Rwanda and Tanzania are made based on Uganda.

#### Americas

A genetic map of the Americas is provided by Salas et al.^48^. Using these data, estimates for Chile, Argentina, and Uruguay are based on Brazil; El Salvador and Guatemala are based on Colombia; Peru is estimated based on the average of Brazil and Colombia.

#### Asia

Genetic clusters for the various Eurasian populations are provided by the GenomeAsia100K Consortium^49^. Using these data, Azerbaijan, Israel, and Turkey are estimated based on the average of Iran and Russia.

#### Europe

A genetic map of Europe is available at: https://www.eupedia.com/europe/european_y-dna_haplogroups_by_region.shtml. The map is based on converging genetic data on the Y-chromosome prevalence from fourteen peer-reviewed, scientific sources ^e.g.,^^50,51^. Based on these data, and coupled also with genetic cluster data from the GenomeAsia100K Consortium^49^, STin2 estimates for Europe are as follows: Austria is estimated based on the average of Hungary and the Czech Republic; Estonia, Finland, and Ukraine are estimated based on Russia; Denmark and Norway are estimated based on Sweden; Poland is estimated based on the average of Czech Republic, Hungary, and Russia; Slovenia is estimated based on Croatia; Switzerland is estimated based on the average of Germany and France; Ireland is estimated based on the UK.

#### Exceptions

One exception to the use of these genetic maps is New Zealand. The related genetic map^49^ detailed the DNA analysis of the local Maori population. However, the native Maori are a minority in New Zealand; 70% of the country’s population is of European (mostly British) descent. Therefore, we estimated New Zealand based on the UK.

### S-allele percentages

S-allele percentages for most countries are provided by Minkov et al.^12^, excepting Croatia and Portugal. The S-allele percentage for Croatia was calculated by averaging data from available studies^56–58^. Data for Portugal are provided by Ferreira et al.^59^.

### Environmental control variables

Conceptual advancements in ecological psychology^18^ have introduced a typology of four spatial and temporal explanations that are theoretically linked to variation in human behavior across countries. First, geographical explanations based on human biogeographical theory suggest that peoples’ habits are dependent on their habitat locations^60^. Second, ecological explanations based on climatic theories of human behavior ^e.g.,^^61^ suggest that climatic harshness shapes peoples’ behaviors as a function of the number of resources they may have to use in coping with these threats coupled with threat severity. Third, evolutionary explanations drawing upon life-history theory ^e.g.,^^62^ suggest that dangerous and unpredictable environments promote fast life strategies (e.g., risk aversion), whereas stable and safe environments promote more self-control. Fourth, contemporary explanations draw upon compensatory-control theories of human behavior ^e.g.,^^63^, suggesting that threat adaptation is a function of the amount of material resources that people have. Thus, we control for variables subsuming all four sets of environmental factors.

#### Geographical controls

First, we control for two geographical factors including latitude and axial orientation. Latitudinal position is defined as the geographical position of a country on a bipolar axis ranging from the South Pole through the equator to the North Pole; scores are available for all countries from van de Vliert and van Lange^18^. Latitude has been found to robustly predict cultural values such as societal individualism, dwarfing associations between values and the concomitant longitudinal position^18^. Axial orientation, defined as the extent to which countries spread out in an east–west vs. a north–south fashion, drives cultural diversity because flora and fauna are more easily domesticated across longitudes than latitudes, allowing for better cultural spread^64^. It has been found to be strongly associated with cultural diversity, even after considering other environmental factors; scores for all non-archipelago countries are provided by Laitin et al.^44^. Data were unavailable on this index for two countries in our study, Japan and New Zealand, because they are archipelagos.

#### Ecological controls

Second, we control for two ecological factors including climatic demands and rainfall steadiness. Climatic demands are defined as a country’s absolute deviation from ambient temperature (22 °C) in both heat and cold in both the hottest and coldest months. It represents the bipolarity of a climate, with larger seasonal variations having greater impacts on human behavior^18^. Climatic demands have been found to robustly predict cultural values such as societal collectivism^45,61^. Rainfall steadiness is defined as the minimum monthly precipitation divided by the maximum monthly precipitation^18^. Scores for all countries are available from van de Vliert and van Lange^18^. It represents year-round water accessibility, a vital resource for human populations, with more steady rainfall leading to reduced threats of both deluge and drought and is theorized to be an effective proxy for climate-induced calamities, such as food scarcity, flooding, soil erosion, and crop ruination^18^.

#### Evolutionary controls

Third, we control for four evolutionary factors including pathogen prevalence (historical and contemporary), social diversity (including racial/ethnic, linguistic, and religious diversity), modernization (including urbanization and population density), and social conflict.

Pathogen prevalence is calculated as the average rate of nine pathogens that capture all biological detriments to human fitness resulting in disease including leishmanias, trypanosomes, malaria, schistosomes, filariae, leprosy, dengue, typhus, and tuberculosis. Both historical and contemporary epidemiological indicators of these variables were calculated because they have both been found to be strongly associated with cultural values such as societal individualism^42^. Scores for all countries are available from Fincher et al.^42^. Data on pathogen prevalence were not available for a few countries in our analysis and were thus estimated based on neighboring countries: Azerbaijan was estimated based on Iran; Rwanda and Uganda were estimated based on Tanzania; Ukraine was estimated based on Russia. Because both historical and contemporary pathogen prevalence were very strongly and positively correlated in our data (*r* = 0.81), an “average pathogen prevalence” measure was calculated for inclusion in analyses.

Social differences in language, race, and religion have a long history of being associated with culture ^e.g.,^^65–67^. We thus control for social diversity, defined as the extent to which a country is fractionalized along ethnic/linguistic/religious lines, and using country scores provided for all countries from Luiz^68^. Because these three types of diversity tend to coincide (e.g., more ethnicities imply more languages spoken), we calculated an averaged “social diversity” score based on the three indices for inclusion in all analyses (*α* = 0.70).

Likewise, modernization theory has long attributed cultural differences to the shift from agricultural and relatively isolated forms of living to urbanized and crowded forms of living^69,70^. We thus control for this factor using World Bank data on the urbanization rate within countries, defined as the percentage of people living in cities^71^, and population density, defined as the logged number of inhabitants per square kilometer in each country^72^. Both these variables have been found to predict cultural values such as collectivism and tightness^43,45,73^.

Finally, social conflict has long been identified as a source of threat mandating the formation of socially cohesive societies^8,74^. More specifically, a country’s history of territorial threat is defined as the number of conflict incidences that have occurred within its territorial borders^8^. Illustratively, the bombing of Pearl Harbor and the Russian Revolution were conflict incidences occurring within US and Russian territorial borders, respectively, whereas the Iraq wars and the invasion of Afghanistan were extra-territorial conflict incidences involving these countries. Data on historical conflict incidences from 1918 to 2016 are available for all countries from the International Crisis Behavior Database^75,76^.

#### Contemporary controls

Fourth, we control for two contemporary factors, including material resources available for threat adaptation both between- and within-countries (i.e., wealth). Wealth differences have been found to strongly predict cultural values such as individualism-collectivism^45,46,61^. Wealth differences between countries is defined as the gross domestic product per capita (GDP PPP); data were available for all countries in our study from the World Bank^77^. Wealth differences within countries is defined as the Gini coefficient; data were available for all countries in our study from the World Bank^78^.

### Dimensions of societal culture

Individualism and monumentalism country scores were available for 50 out of 55 countries in our study^20^. The remaining five countries were estimated based on cultural values clusters across world regions^10^, as follows: Burkina Faso and Zambia were based on South Africa; El Salvador was based on Colombia; Tanzania and Uganda were based on Rwanda.

## Supplementary Information

Supplementary Information.

## Data Availability

The data used in this study are available from the following sources: **STin2**: Please see Table S2 below for the 75 primary studies that these data were extracted from. **S-allele (Croatia)**: Hranilovic et al.^56^. Noskova et al.^57^. Božina et al.^58^. **S-allele (Portugal)**: Ferreira et al.^59^. **S-allele (All Other Countries)**: Minkov et al.^12^. **Latitudinal position, climatic demands, rainfall steadiness, historical pathogen prevalence, and contemporary pathogen prevalence**: Van de Vliert and van Lange^18^. **Axial orientation**: Laitin et al.^44^. **Social diversity including racial/ethnic, religious, and linguistic diversity**: Luiz^68^. **Urbanization rate**: World Bank^71^. **Population density**: World Bank^72^. **GDP PPP**: World Bank^77^. **Gini coefficient**: World Bank^78^. **Number of territorial conflicts**: Brecher and Wilkenfeld^75^. Brecher et al.^76^. **Cultural dimensions**: Minkov and Kaasa^20^.
