# Supplementary material for: Genetic predictors of cultural values variation between societies
Source: Sci Rep. 2023 May 17;13:7986. doi: 10.1038/s41598-023-34845-x (PMC10192116; doi:10.1038/s41598-023-34845-x)
Supplement: Supplementary file 1 — Supplementary Information. [file 41598_2023_34845_MOESM1_ESM.docx]

**Table S1**

*STin2 Percentages by Country*

| **Country** | **% 12/12** | **% 10/10** | **Source** |
| --- | --- | --- | --- |
| Australia | 30.94 | 18.71 | a |
| Belgium | 40.31 | 12.63 | a |
| Brazil | 39.49 | 23.21 | a |
| Canada | 31.10 | 16.14 | a |
| China | 79.88 | 1.45 | a |
| Colombia | 44.67 | 13.33 | a |
| Croatia | 37.56 | 16.17 | a |
| Czech Republic | 39.04 | 15.11 | a |
| France | 36.36 | 18.18 | a |
| Germany | 25.90 | 10.26 | a |
| Hungary | 37.35 | 15.11 | a |
| India | 51.89 | 9.57 | a |
| Iran | 42.50 | 10.50 | a |
| Italy | 40.00 | 9.23 | a |
| Japan | 79.15 | 1.42 | a |
| Netherlands | 40.56 | 14.78 | a |
| Portugal | 38.17 | 14.59 | a |
| Russia | 35.53 | 19.52 | a |
| Singapore | 85.37 | 7.32 | a |
| South Africa | 60.00 | 4.48 | a |
| South Korea | 83.86 | 0.98 | a |

**Table S1**

*(Continued)*

| **Country** | **% 12/12** | **% 10/10** | **Source** |
| --- | --- | --- | --- |
| Spain | 44.18 | 13.11 | a |
| Sweden | 29.09 | 9.09 | a |
| Taiwan | 79.83 | 1.68 | a |
| Uganda | 57.22 | 9.93 | a |
| UK | 35.18 | 15.42 | a (England and Scotland) |
| USA | 43.02 | 11.80 | a |
| Vietnam | 74.26 | 2.31 | a |
| Argentina | 39.49 | 23.21 | Estimate based on Brazil |
| Austria | 38.20 | 15.11 | Estimate based on Czech Republic & Hungary |
| Azerbaijan | 39.02 | 15.01 | Estimate based on Iran & Russia |
| Burkina Faso | 60.00 | 4.48 | Estimate based on South Africa |
| Chile | 39.49 | 23.21 | Estimate based on Brazil |
| Denmark | 29.09 | 9.09 | Estimate based on Sweden |
| El Salvador | 44.67 | 13.33 | Estimate based on Colombia |
| Estonia | 35.53 | 19.52 | Estimate based on Russia |
| Finland | 35.53 | 19.52 | Estimate based on Russia |
| Ghana | 60.00 | 4.48 | Estimate based on South Africa |
| Guatemala | 44.67 | 13.33 | Estimate based on Colombia |
| Ireland | 35.18 | 15.42 | Estimate based on UK |
| Israel | 39.02 | 15.01 | Estimate based on Iran & Russia |

**Table S1**

*(Continued)*

| **Country** | **% 12/12** | **% 10/10** | **Source** |
| --- | --- | --- | --- |
| New Zealand | 35.18 | 15.42 | Estimate based on UK |
| Norway | 29.09 | 9.09 | Estimate based on Sweden |
| Peru | 42.08 | 18.27 | Estimate based on Brazil & Colombia |
| Poland | 37.31 | 16.58 | Estimate based on Czech Republic, Hungary & Russia |
| Rwanda | 57.22 | 9.93 | Estimate based on Uganda |
| Slovenia | 37.56 | 16.17 | Estimate based on Croatia |
| Switzerland | 31.13 | 14.22 | Estimate based on France & Germany |
| Tanzania | 57.22 | 9.93 | Estimate based on Uganda |
| Turkey | 39.02 | 15.01 | Estimate based on Iran & Russia |
| Ukraine | 35.53 | 19.52 | Estimate based on Russia |
| Uruguay | 39.49 | 23.21 | Estimate based on Brazil |
| Zambia | 60.00 | 4.48 | Estimate based on South Africa |
| Zimbabwe | 60.00 | 4.48 | Estimate based on South Africa |

*Note: “a” indicates that primary data were available, as displayed in Table S1.*

**Table S2**

*Studies Included in the STin2 Genetic Database*

| **Country** | **Authors** | **Year** | **Sample Type** | ***N*** | ***n* 12/12** | ***n* 10/10** |
| --- | --- | --- | --- | --- | --- | --- |
| Australia | Stjepanovic ́ et al.^79^ | 2013 | Clinical | 139 | 43 | 26 |
| Belgium | Van Assche et al.^80^ | 2016 | Non-clinical | 1037 | 418 | 131 |
| Brazil | de Castro Pizzo et al.^81^ | 2014 | Non-clinical  Clinical | 175  185 | 72  82 | 49  26 |
| Brazil | de Castro Pizzo et al.^82^ | 2015 | Non-clinical  Clinical | 112  247 | 50  104 | 29  46 |
| Brazil | de Lima et al.^83^ | 2012 | Non-clinical  Clinical | 126  60 | 17  10 | 88  31 |
| Brazil | Gomes et al.^84^ | 2018 | Non-clinical  Clinical | 197  197 | 84  95 | 31  17 |
| Brazil | Kohlrausch et al.^85^ | 2010 | Clinical | 116 | 44 | 15 |
| Brazil | Miguita et al.^86^ | 2011 | Clinical | 41 | 17 | 6 |
| Canada | Lopez de Lara et al.^87^ | 2006 | Non-clinical  Clinical | 152  102 | 58  21 | 22  19 |
| China | Huang et al.^88^ | 2016 | Non-clinical  Clinical | 101  114 | 57  81 | 6  6 |
| China | Li et al.^89^ | 2007 | Clinical | 586 | 494 | 1 |
| China | Lin et al.^90^ | 2009 | Non-clinical  Clinical | 282  140 | 184  91 | 9  6 |
| China | Min et al.^91^ | 2009 | Non-clinical  Clinical | 437  579 | 368  480 | 3  6 |
| China  China | Wang et al.^92^  Zhong et al.^93^ | 2016  2012 | Non-clinical  Clinical  Non-Clinical  Clinical | 199  200  231  233 | 167  172  190  194 | 1  0  2  5 |

**Table S2**

*(Continued)*

| **Country** | **Authors** | **Year** | **Sample Type** | ***N*** | ***n* 12/12** | ***n* 10/10** |
| --- | --- | --- | --- | --- | --- | --- |
| Colombia | Perea et al.^94^ | 2012 | Non-clinical | 300 | 134 | 40 |
| Croatia  Croatia | Blazevic et al.^16^  Grubelic et al.^95^ | 2017  2018 | Non-clinical  Non-clinical  Clinical | 50  157  192 | 11  55  76 | 6  35  26 |
| Czech Republic | Kitzlerová et al.^96^ | 2018 | Non-clinical  Clinical | 90  68 | 26  30 | 15  12 |
| Czech Republic | Slezakova et al.^97^ | 2020 | Non-clinical  Clinical | 153  86 | 60  39 | 21  12 |
| England | Niesler et al.^98^ | 2010 | Non-clinical  Clinical | 92  196 | 32  70 | 13  31 |
| France | Betancur et al.^99^ | 2002 | Clinical | 44 | 16 | 8 |
| Germany | Kaiser et al.^100^ | 2001 | Non-clinical  Clinical | 587  684 | 47  259 | 25  95 |
| Germany | Tadić et al.^101^ | 2010 | Non-clinical  Clinical | 152  156 | 56  47 | 17  25 |
| Hungary | Fehér et al.^102^ | 2013 | Non-clinical  Clinical | 234  252 | 79  98 | 47  38 |
| Hungary | Sarosi et al.^103^ | 2008 | Non-clinical  Clinical | 30  71 | 11  27 | 3  17 |
| Hungary | Szilagyi et al.^104^ | 2006 | Non-clinical  Clinical | 464  87 | 172  38 | 59  8 |
| India | Banerjee et al.^105^ | 2006 | Non-clinical  Clinical | 276  56 | 139  33 | 26  4 |
| India | Guhathakurta et al.^106^ | 2008 | Non-clinical  Clinical | 310  83 | 177  45 | 17  4 |

**Table S2**

*(Continued)*

| **Country** | **Authors** | **Year** | **Sample Type** | ***N*** | ***n* 12/12** | ***n* 10/10** |
| --- | --- | --- | --- | --- | --- | --- |
| India | Jaiswal et al.^107^ | 2015 | Non-clinical  Clinical | 485  169 | 261  86 | 40  13 |
| India | Joshi et al.^27^ | 2010 | Non-clinical  Clinical | 217  217 | 109  110 | 24  35 |
| India | Pasi et al.^108^ | 2015 | Non-clinical | 39 | 23 | 4 |
| India | Sahni et al.^109^ | 2019 | Non-clinical  Clinical | 109  141 | 65  85 | 7  11 |
| India | Tharoor et al.^110^ | 2013 | Non-clinical  Clinical | 124  122 | 59  60 | 13  13 |
| India | Vijayan et al.^26^ | 2009 | Non-clinical  Clinical | 243  240 | 90  127 | 36  24 |
| Iran | Farjadian et al.^111^ | 2018 | Non-clinical  Clinical | 100  100 | 41  44 | 13  8 |
| Italy | Terrazzino et al.^112^ | 2010 | Clinical | 130 | 52 | 12 |
| Japan | Ito et al.^113^ | 2002 | Clinical | 54 | 39 | 1 |
| Japan | Ohara et al.^114^ | 1999 | Clinical | 103 | 89 | 1 |
| Japan | Takahashi et al.^115^ | 2002 | Clinical | 54 | 39 | 1 |
| Netherlands | Smits et al.^116^ | 2007 | Clinical | 183 | 80 | 30 |
| Netherlands | Smits et al.^117^ | 2008 | Non-clinical  Clinical | 162  50 | 70  22 | 25  11 |
| Netherlands | Quaak et al.^118^ | 2012 | Non-clinical | 214 | 75 | 24 |
| Portugal  Portugal | Dias et al.^119^  Manco et al.^120^ | 2016  2020 | Clinical  Clinical | 523  567 | 197  219 | 87  72 |
| Russia | Gaysina et al.^121^ | 2006 | Non-clinical  Clinical | 244  144 | 95  49 | 43  24 |

**Table S2**

*(Continued)*

| **Country** | **Authors** | **Year** | **Sample Type** | ***N*** | ***n* 12/12** | ***n* 10/10** |
| --- | --- | --- | --- | --- | --- | --- |
| Russia  Scotland  Singapore | Toshchakova et al.^122^  Ogilvie et al.^123^  Zhong et al.^95^ | 2017  1996  2012 | Non-clinical  Clinical  Non-clinical  Clinical  Non-clinical | 161  194  193  83  41 | 46  74  65  26  35 | 38  40  28  18  3 |
| South Africa | Hemmings et al.^17^ | 2018 | Clinical | 290 | 174 | 13 |
| South Korea | Choi-Kwon et al.^124^ | 2013 | Clinical | 508 | 426 | 5 |
| Spain | Baca-Garcia et al.^125^ | 2007 | Non-clinical  Clinical | 406  675 | 176  298 | 50  76 |
| Spain | Florez et al.^126^ | 2008 | Clinical | 90 | 37 | 13 |
| Spain | Saiz et al.^31^ | 2008 | Non-clinical  Clinical | 420  508 | 174  257 | 64  71 |
| Spain | Saiz et al.^35^ | 2008 | Non-clinical  Clinical | 420  193 | 174  82 | 64  30 |
| Spain | Saiz et al.^127^ | 2009 | Non-clinical  Clinical | 420  278 | 174  124 | 64  40 |
| Spain | Saiz et al.^128^ | 2010 | Clinical | 404 | 169 | 62 |
| Spain | Sanjuan et al.^129^ | 2008 | Clinical | 1084 | 499 | 108 |
| Sweden | Bah et al.^33^ | 2008 | Non-clinical  Clinical | 9  9 | 5  5 | 0  1 |
| Sweden | de Mel et al.^130^ | 2012 | Non-clinical | 32  60 | 8  14 | 1  8 |
| Taiwan | Kao et al.^131^ | 2018 | Clinical | 119 | 95 | 2 |
| Uganda | Kalungi et al.^25^ | 2017 | Clinical | 554 | 317 | 55 |
| USA | Jarrett et al.^132^ | 2007 | Clinical | 138 | 55 | 20 |
| USA | Jasinska et al.^133^ | 2012 | Non-clinical | 82 | 32 | 12 |

**Table S2**

*(Continued)*

| **Country** | **Authors** | **Year** | **Sample Type** | ***N*** | ***n* 2/12** | ***n* 10/10** |
| --- | --- | --- | --- | --- | --- | --- |
| USA  USA  USA | Kohen et al.^30^  Kohen et al.^28^  Mercer et al.^134^ | 2008  2009  2012 | Non-clinical  Clinical  Non-clinical  Clinical  Clinical | 75  75  48  186  204 | 24  43  19  74  75 | 12  7  6  27  26 |
| USA | Mitchell et al.^34^ | 2011 | Clinical | 1206 | 622 | 105 |
| USA | Payer et al.^135^ | 2012 | Non-clinical  Clinical | 47  53 | 23  23 | 6  9 |
| USA | Shiroma et al.^136^ | 2014 | Clinical | 216 | 80 | 34 |
| USA | Sulik et al.^137^ | 2012 | Non-clinical | 245 | 84 | 25 |
| USA | Taylor et al.^138^ | 2014 | Non-clinical | 340 | 154 | 29 |
| USA  USA | Wendland et al.^55^  Yohannes et al.^139^ | 2007  2020 | Non-clinical  Clinical  Clinical | 657  295  294 | 256  108  118 | 88  52  33 |
| Vietnam | Koks et al.^29^ | 2018 | Non-clinical | 369  1453 | 268  1085 | 8  34 |

**Table S3** *Means, Standard Deviations, and Intercorrelations between Study Variables.*

| Variable | *M* | *SD* | 1 | 2 | 3 | 4 | 5 | 6 | 7 | 8 | 9 | 10 | 11 | 12 | 13 | 14 | 15 | 16 |
| --- | --- | --- | --- | --- | --- | --- | --- | --- | --- | --- | --- | --- | --- | --- | --- | --- | --- | --- |
| 1. % S-allele | 45.24 | 14.55 | ----- |  |  |  |  |  |  |  |  |  |  |  |  |  |  |  |
| 2. % STin2 12/12 | 45.88 | 15.21 | .30 | ----- |  |  |  |  |  |  |  |  |  |  |  |  |  |  |
| 3. % STin2 10/10 | 12.50 | 6.07 | -.08 | -.75 | ----- |  |  |  |  |  |  |  |  |  |  |  |  |  |
| 4. Individualism | -12.78 | 96.28 | .22 | -.52 | .26 | ----- |  |  |  |  |  |  |  |  |  |  |  |  |
| 5. Monumentalism | -23.48 | 103.75 | .58 | .07 | -.08 |  | ----- |  |  |  |  |  |  |  |  |  |  |  |
| 6. Latitude | 24.34 | 29.27 | .12 | -.31 | -.00 | .52 |  | ----- |  |  |  |  |  |  |  |  |  |  |
| 7. Axial Orientation | 1.18 | 0.84 | .16 | -.06 | .18 | .07 | -.03 |  | ----- |  |  |  |  |  |  |  |  |  |
| 8. Climatic Demands | 65.10 | 19.93 | .09 | -.52 | .31 | .67 | .49 | -.10 |  | ----- |  |  |  |  |  |  |  |  |
| 9. Rainfall Steadiness | 0.27 | 0.23 | .02 | -.46 | .49 | .70 | .44 | -.16 | .47 |  | ----- |  |  |  |  |  |  |  |
| 10. Pathogen Prevalence | 16.08 | 3.78 | -.18 | .49 | -.34 | -.86 | -.61 | .10 | -.65 | -.69 |  | ----- |  |  |  |  |  |  |
| 11. Social Diversity | 0.39 | 0.20 | -.42 | .32 | -.32 | -.50 | -.31 | -.21 | -.32 | -.22 | .44 |  | ----- |  |  |  |  |  |
| 12. Log Population Density | 278 | 1019 | .28 | .42 | -.18 | -.02 | .19 | -.11 | -.26 | .19 | -.10 | .06 |  | ----- |  |  |  |  |
| 13. Urbanization Rate | 68.70 | 20.66 | .43 | -.30 | .37 | .62 | .38 | .20 | .32 | .52 | -.58 | -.43 | .19 |  | ----- |  |  |  |
| 14. GDP PPP | 24791 | 23689 | .16 | -.34 | .10 | .78 | .55 | .04 | .44 | .66 | -.76 | -.32 | .22 | .56 |  | ----- |  |  |
| 15. Gini Coefficient | 37.89 | 9.67 | -.02 | .19 | .10 | -.48 | -.59 | .20 | -.52 | -.31 | .50 | .19 | .02 | .05 | -.43 |  | ----- |  |
| 16. Territorial Conflicts | 5.52 | 5.97 | .27 | .18 | -.20 | -.11 | .21 | -.01 | .17 | -.32 | .13 | -.02 | -.07 | .14 | -.21 | -.11 |  | ----- |

*N = 55 countries; Correlations at or above |.26| are significant at α = .05; correlations at or above |.33| are significant at α = .01; Pathogen prevalence is reported as the average of historical and contemporary pathogen prevalence.*

**Table S4**: *Robustness Tests – Excluding Countries with Small Sample Sizes on STin2*

| **Independent variables** | **Societal cultural dimensions** | | |
| --- | --- | --- | --- |
|  | Individualism | Monumentalism |  |
| Step 1: Control Variables ^a^ |  |  |  |
| Latitude | -.025 | **.262^*^** |  |
| Axial orientation | -.013 | .028 |  |
| Climatic demands | .048 | .212 |  |
| Rainfall steadiness | .134 | **.265^+^** |  |
| Pathogen prevalence average | **-.380^+^** | .035 |  |
| Social diversity | -.055 | .117 |  |
| Logged population density | .073 | .033 |  |
| Urbanization rate | .129 | .057 |  |
| GDP PPP | **.259^+^** | .204 |  |
| Gini coefficient | -.109 | **-.327^**^** |  |
| Number of Territorial Conflicts | -.022 | .117 |  |
| *Adjusted R²* | .788 | .670 |  |
| *F* | **16.907^***^** | **9.680^***^** |  |
| Step 2: 5-HTTLPR |  |  |  |
| % S-allele | .127 | **.423^***^** |  |
| *Adjusted R²* | .792 | .770 |  |
| *∆ R²* | .004 | .100 |  |
| *F* | 1.666 | **16.702^***^** |  |
| Step 3: STin2 added |  |  |  |
| % 12/12 | .037 | **.443^*^** |  |
| % 10/10 | .055 | .217 |  |
| *Adjusted R²* | .780 | .803 |  |
| *∆ R²* | .000 | .033 |  |
| *F* | .060 | **3.898^*^** |  |

^a^ *Standardized regression coefficients for the control variables are from Step 3; n = 48 countries*

*Note: S-allele percentage effects are displayed for both Steps 2 and 3 to facilitate comparisons on the effects of 5-HTTLPR both with and without consideration of STin2.*

*+ p* < .10; * *p* < .05; ** *p* < .01; *** *p* < .001

**Table S5:** *Robustness Tests – Excluding Countries with Estimated Values on Both Predictors and Criteria*

| **Independent variables** | **Societal cultural dimensions** | | |
| --- | --- | --- | --- |
|  | Individualism | Monumentalism |  |
| Step 1: Control Variables ^a^ |  |  |  |
| Latitude | -.017 | **.287^*^** |  |
| Axial orientation | -.055 | .001 |  |
| Climatic demands | -.102 | .070 |  |
| Rainfall steadiness | .116 | **.244^+^** |  |
| Pathogen prevalence average | **-.526^**^** | -.111 |  |
| Social diversity | -.107 | .082 |  |
| Logged population density | **-.209^+^** | -.156 |  |
| Urbanization rate | .145 | .114 |  |
| GDP PPP | **.240^+^** | .157 |  |
| Gini coefficient | -.102 | **-.351^**^** |  |
| Number of Territorial Conflicts | .042 | .112 |  |
| *Adjusted R²* | .793 | .526 |  |
| *F* | **17.734^***^** | **5.840^***^** |  |
| Step 2: 5-HTTLPR |  |  |  |
| % S-allele | **.148^+^** | **.536^***^** |  |
| *Adjusted R²* | .804 | .725 |  |
| *∆ R²* | .011 | .199 |  |
| *F* | **2.968^+^** | **27.587^***^** |  |
| Step 3: STin2 added |  |  |  |
| % 12/12 | .015 | **.587^**^** |  |
| % 10/10 | .042 | .200 |  |
| *Adjusted R²* | .793 | .799 |  |
| *∆ R²* | .000 | .074 |  |
| *F* | .060 | **7.549^**^** |  |

^a^ *Standardized regression coefficients for the control variables are from Step 3; n = 49 countries*

*Note: S-allele percentage effects are displayed for both Steps 2 and 3 to facilitate comparisons on the effects of 5-HTTLPR both with and without consideration of STin2.*

*+ p* < .10; * *p* < .05; ** *p* < .01; *** *p* < .001

**Table S6**

*Robustness Tests – Only Countries with Primary Data on STin2*

| **Independent variables** | **Societal cultural dimensions** | |
| --- | --- | --- |
|  | Individualism | Monumentalism |
| Step 1: Control Variables ^a^ |  |  |
| Latitude | .081 | .011 |
| Axial orientation | .017 | **.244^+^** |
| Climatic demands | .061 | .087 |
| Rainfall steadiness | .004 | -.048 |
| Pathogen prevalence average | .204 | **-.448^+^** |
| Social diversity | -.200 | .097 |
| Logged population density | -.224 | **-.341^+^** |
| Urbanization rate | **.383^+^** | .034 |
| GDP PPP | **.568^*^** | **.547^*^** |
| Gini coefficient | **-.423^+^** | -.119 |
| Number of Territorial Conflicts | .086 | **.374^*^** |
| *Adjusted R²* | .781 | .358 |
| *F* | **9.431^***^** | **2.317^+^** |
| Step 2: 5-HTTLPR |  |  |
| % S-allele | -.224 | **.696**** |
| *Adjusted R²* | .797 | .619 |
| *∆ R²* | .016 | .261 |
| *F* | 2.196 | **11.257**** |
| Step 3: STin2 added |  |  |
| % 12/12 | .273 | **1.138^***^** |
| % 10/10 | .112 | **.711^**^** |
| *Adjusted R²* | .781 | .847 |
| *∆ R²* | .000 | .228 |
| *F* | .483 | **11.471**** |

^a^ *Standardized regression coefficients for the control variables are from Step 3; n = 27 countries*

*Note: S-allele percentage effects are displayed for both Steps 2 and 3 to facilitate comparisons on the effects of 5-HTTLPR both with and without consideration of STin2.*

*+ p* < .10; * *p* < .05; ** *p* < .01; *** *p* < .001

**Supplementary References**

*All studies with primary data on STin2 are referenced below.*

1. Blazevic, S. *et al.* Epigenetic adaptation of the placental serotonin transporter gene (SLC6A4) to gestational diabetes mellitus. *PloS One* ***12***, e0179934; <https://doi.org/10.1371/journal.pone.0179934> (2017).
2. Hemmings, S. *et al.* Appetitive and reactive aggression are differentially associated with the STin2 genetic variant in the serotonin transporter gene. *Sci. Rep*. ***8***, 6714, 2-3; <https://doi.org/10.1038/s41598-018-25066-8> (2018).
3. Kalungi, A. *et al.* Association between serotonin transporter gene polymorphisms and increased suicidal risk among HIV positive patients in Uganda. *BMC Genet.* ***18***, 71, 2-7; <https://doi.org/10.1186/s12863-017-0538-y> (2017).
4. Vijayan, N. N. *et al.* Evidence of association of serotonin transporter gene polymorphisms with schizophrenia in a South Indian population. *J. Hum. Genet*. ***54***, 538–542; <https://doi.org/10.1038/jhg.2009.76> (2009).
5. Joshi, G., Pradhan, S., & Mittal, B. No direct association of serotonin transporter (STin2 VNTR) and receptor (HT 102T>C) gene variants in genetic susceptibility to migraine. *Dis. Markers*. ***29***, 223–229; <https://doi.org/10.3233/DMA-2010-0752> (2010).
6. Kohen, R., Jarrett, M. E., Cain, K. C., Jun, S. E., Navaja, G. P., Symonds, S., & Heitkemper, M. M. The serotonin transporter polymorphism rs25531 is associated with irritable bowel syndrome. *Dig. Dis. Sci*. ***54***, 2663–2670; <https://doi.org/10.1007/s10620-008-0666-3> (2009).
7. Koks, G. *et al.* Genetic interaction between two VNTRs in the SLC6A4 gene regulates nicotine dependence in Vietnamese men. *Front. Pharmacol*. ***9***, 1398, 1-8; <https://doi.org/10.3389/fphar.2018.01398> (2018).
8. Kohen, R. *et al.* Association of serotonin transporter gene polymorphisms with poststroke depression. *Arch. Gen. Psychiatry*. ***65***, 1296–1302; <https://doi.org/10.1001/archpsyc.65.11.1296> (2008).
9. Saiz, P. A. *et al.* Association between the A-1438G polymorphism of the serotonin 2A receptor gene and nonimpulsive suicide attempts. *Psychiatr. Genet*. ***18***, 213–218; <https://doi.org/10.1097/YPG.0b013e3283050ada> (2008).
10. Bah, J. *et al.* Serotonin transporter gene polymorphisms: effect on serotonin transporter availability in the brain of suicide attempters. *Psychiatry Res*. ***162***, 221–229; <https://doi.org/10.1016/j.pscychresns.2007.07.004> (2008).
11. Mitchell, C. *et al.* Role of mother's genes and environment in postpartum depression. *Proc. Natl. Acad. Sci*. ***108***, 8189–8193; <https://doi.org/10.1073/pnas.1014129108> (2011).
12. Saiz, P. A. *et al.* Association study between obsessive-compulsive disorder and serotonergic candidate genes. *Prog. Neuropsychopharmacol. Biol. Psychiatry*. ***32***, 765–770; <https://doi.org/10.1016/j.pnpbp.2007.12.005> (2008).
13. Wendland, J. R., Kruse, M. R., Cromer, K. R., & Murphy, D. L. A large case-control study of common functional SLC6A4 and BDNF variants in obsessive-compulsive disorder. *Neuropharmacol.* ***32***, 2543–2551; <https://doi.org/10.1038/sj.npp.1301394> (2007).
14. Stjepanović, D., Lorenzetti, V., Yücel, M., Hawi, Z., & Bellgrove, M. A. Human amygdala volume is predicted by common DNA variation in the stathmin and serotonin transporter genes. *Transl. Psychiatry*. ***3***, e283, 3; <https://doi.org/10.1038/tp.2013.41> (2013).
15. Van Assche, E. *et al.* Depressive symptoms in adolescence: The role of perceived parental support, psychological control, and proactive control in interaction with 5-HTTLPR. *Eur. Psychiatry.* ***35***, 55–63; <https://doi.org/10.1016/j.eurpsy.2016.01.2428> (2016).
16. de Castro Pizzo, M. R. *et al.* SLC6A4 STin2 VNTR genetic polymorphism is associated with tobacco use disorder, but not with successful smoking cessation or smoking characteristics: a case control study. *BMC Genet*. ***15***, 78, 2-8; <https://doi.org/10.1186/1471-2156-15-78> (2014).
17. de Castro Pizzo, M. R. *et al.* STin2 VNTR polymorphism is associated with comorbid tobacco use and mood disorders. *J. Affect. Disord*. ***172***, 347–354; <https://doi.org/10.1016/j.jad.2014.10.023> (2015).
18. de Lima, K. W. *et al.* Association between the STin2 VNTR polymorphism and smoking behavior in oral cancer patients and healthy individuals. *Clin. Exp. Med.* ***12***, 13–19; <https://doi.org/10.1007/s10238-011-0140-y> (2012).
19. Gomes, C. *et al.* Association analysis of SLC6A4 and HTR2A genes with obsessive-compulsive disorder: Influence of the STin2 polymorphism. *Compr. Psychiatry*. ***82***, 1–6; <https://doi.org/10.1016/j.comppsych.2017.12.004> (2018).
20. Kohlrausch, F. B. *et al.* Influence of serotonin transporter gene polymorphisms on clozapine response in Brazilian schizophrenics. *J. Psychiatr. Res.* ***44***, 1158–1162; <https://doi.org/10.1016/j.jpsychires.2010.04.003> (2010).
21. Miguita, K., Cordeiro, Q., Shavitt, R. G., Miguel, E. C., & Vallada, H. Association study between genetic monoaminergic polymorphisms and OCD response to clomipramine treatment. *Arq. Neuro-Psiquiatr.* ***69***, 283–287; <https://doi.org/10.1590/s0004-282x2011000300003> (2011).
22. Lopez de Lara, C. *et al.* STin2 variant and family history of suicide as significant predictors of suicide completion in major depression. *Biol. Psychiatry*. ***59***, 114–120; <https://doi.org/10.1016/j.biopsych.2005.06.021> (2006).
23. Huang, Y. *et al.* Association of STin2 VNTR polymorphism of serotonin transporter gene with lifelong premature ejaculation: A case-control study in Han Chinese subjects. *Med. Sci. Monit.* ***22***, 3588–3594; <https://doi.org/10.12659/msm.897720> (2016).
24. Li, J. Z., Wang, Y., Zhou, R., Zhang, H., Yang, L., Wang, B., & Faraone, S. V. Association between polymorphisms in serotonin transporter gene and attention deficit hyperactivity disorder in Chinese Han subjects. *Am. J. Med. Genet. B. Neuropsychiatr. Genet.* ***144B***, 14–19; <https://doi.org/10.1002/ajmg.b.30373> (2007).
25. Lin, C. *et al.* Haplotype analysis confirms association of the serotonin transporter (5-HTT) gene with schizophrenia in the Han Chinese population. *Neurosci. Lett.* ***453***, 210–213; <https://doi.org/10.1016/j.neulet.2009.02.023> (2009).
26. Min, W. *et al.* Monoamine transporter gene polymorphisms affect susceptibility to depression and predict antidepressant response. J. *Psychopharmacol.* ***205***, 409–417; <https://doi.org/10.1007/s00213-009-1550-3> (2009).
27. Wang, Y. *et al.* Psychosocial mechanisms of serotonin transporter's genetic polymorphism in susceptibility to major depressive disorder: mediated by trait coping styles and interacted with life events. *Am. J. Transl. Res.* ***8***, 1281–1292; <https://www.ncbi.nlm.nih.gov/pmc/articles/PMC4846972/> (2016).
28. Zhong, S., Chark, R., Ebstein, R. P., & Chew, S. H. Imaging genetics for utility of risks over gains and losses. *NeuroImage* ***59***, 540–546; <https://doi.org/10.1016/j.neuroimage.2011.07.031> (2012).
29. Perea, C. S., Paternina, A. C., Gomez, Y., & Lattig, M. C. Negative affectivity moderated by BDNF and stress response. *J. Affect. Disord.* ***136***, 767–774; <https://doi.org/10.1016/j.jad.2011.09.043> (2012).
30. Grubelic, R. K. *et al.* Association of polymorphic variants in serotonin re-uptake transporter gene with Crohn's disease: a retrospective case-control study. *Croat. Med. J.* ***59***, 232–243; <https://doi.org/10.3325/cmj.2018.59.232> (2018).
31. Kitzlerova, E. *et al.* Interactions among polymorphisms of susceptibility loci for Alzheimer’s disease or depressive disorder. *Med. Sci. Monit.* ***24***, 2599–2619; <https://doi.org/10.12659/MSM.907202> (2018).
32. Slezakova, S. *et al.* Serotonin transporter gene (SLC6A4) variability in patients with recurrent aphthous stomatitis. *Arch. Oral Biol.* ***110***, 104628, 3; <https://doi.org/10.1016/j.archoralbio.2019.104628> (2020).
33. Niesler, B. *et al.* 5-HTTLPR and STin2 polymorphisms in the serotonin transporter gene and irritable bowel syndrome: effect of bowel habit and sex. *J. Gastroenterol.* ***22***, 856–861; <https://doi.org/10.1097/MEG.0b013e32832e9d6b> (2010).
34. Betancur, C. *et al.* Serotonin transporter gene polymorphisms and hyperserotonemia in autistic disorder. *Mol. Psychiatry*. ***7***, 67–71; <https://doi.org/10.1038/sj/mp/4000923> (2002).
35. Kaiser, R. *et al.* Serotonin transporter polymorphisms: No association with response to antipsychotic treatment, but associations with the schizoparanoid and residual subtypes of schizophrenia. *Mol. Psychiatry*. ***6***, 179–185; <https://doi.org/10.1038/sj.mp.4000821> (2001).
36. Tadic, A. *et al.* Association analysis between gene variants of the tyrosine hydroxylase and the serotonin transporter in borderline personality disorder.  *World J. Biol. Psychiatry*. ***11***, 45–58; <https://doi.org/10.3109/15622970903406226> (2010).
37. Feher, A. *et al.* Serotonin transporter and serotonin receptor 2A gene polymorphisms in Alzheimer's disease. *Neurosci. Lett.* ***534***, 233–236; <https://doi.org/10.1016/j.neulet.2012.12.020> (2013).
38. Sarosi, A. *et al.* Association of the STin2 polymorphism of the serotonin transporter gene with a neurocognitive endophenotype in major depressive disorder. *Prog. Neuropsychopharmacol. Biol. Psychiatry.* ***32***, 1667–1672; <https://doi.org/10.1016/j.pnpbp.2008.06.014> (2008).
39. Szilagyi, A. *et al.* Contribution of serotonin transporter gene polymorphisms to pediatric migraine. *Headache* ***46***, 478–485; <https://doi.org/10.1111/j.1526-4610.2006.00379.x> (2006).
40. Banerjee, E. *et al.* A family-based study of Indian subjects from Kolkata reveals allelic association of the serotonin transporter intron-2 (STin2) polymorphism and attention-deficit-hyperactivity disorder (ADHD). *Am. J. Med. Genet. B. Neuropsychiatr. Genet.* ***141B***, 361–366; <https://doi.org/10.1002/ajmg.b.30296> (2006).
41. Guhathakurta, S. *et al.* Population-based association study and contrasting linkage disequilibrium pattern reveal genetic association of SLC6A4 with autism in the Indian population from West Bengal. *Brain Res.* ***1240***, 12–21; <https://doi.org/10.1016/j.brainres.2008.08.063> (2008).
42. Jaiswal, P. *et al.* SLC6A4 markers modulate platelet 5-HT level and specific behaviors of autism: a study from an Indian population. *Prog. Neuropsychopharmacol. Biol. Psychiatry.* ***56***, 196–206; <https://doi.org/10.1016/j.pnpbp.2014.09.004> (2015).
43. Pasi, S. *et al.* Evaluation of psychiatric and genetic risk factors among primary relatives of suicide completers in Delhi NCR region, India. *Psychiatry Res*. ***229***, 933–939; <https://doi.org/10.1016/j.psychres.2015.07.015> (2015).
44. Sahni, S. *et al.* Association of serotonin and GABA pathway gene polymorphisms with alcohol dependence: A preliminary study. *Asian J. Psychiatr*. ***39***, 169–173; <https://doi.org/10.1016/j.ajp.2018.04.023> (2019).
45. Tharoor, H., Kotambail, A., Jain, S., Sharma, P. S., & Satyamoorthy, K. Study of the association of serotonin transporter triallelic 5-HTTLPR and STin2 VNTR polymorphisms with lithium prophylaxis response in bipolar disorder. *Psychiatr. Genet*. ***23***, 77–81; <https://doi.org/10.1097/YPG.0b013e32835d6fad> (2013).
46. Farjadian, S. *et al.* Polymorphisms of serotonin transporter gene and psychological status in patients with multiple sclerosis. *Iran. J. Neurol*. ***17***, 105–110; <https://www.ncbi.nlm.nih.gov/pmc/articles/PMC6420687/> (2018).
47. Terrazzino, S. *et al.* The serotonin transporter gene polymorphism STin2 VNTR confers an increased risk of inconsistent response to triptans in migraine patients. *Eur. J. Pharmacol.* ***641***, 82–87; <https://doi.org/10.1016/j.ejphar.2010.04.049> (2010).
48. Ito, K. *et al.* A variable number of tandem repeats in the serotonin transporter gene does not affect the antidepressant response to fluvoxamine. *Psychiatry Res*. ***111***, 235–239; <https://doi.org/10.1016/s0165-1781(02)00141-5> (2002).
49. Ohara, K. *et al.* A variable-number-tandem-repeat of the serotonin transporter gene and anxiety disorders. *Prog. Neuropsychopharmacol. Biol. Psychiatry.* ***23***, 55–65; <https://doi.org/10.1016/s0278-5846(98)00091-8> (1999).
50. Takahashi, H. *et al.* No association between the serotonergic polymorphisms and incidence of nausea induced by fluvoxamine treatment. *Eur. Neuropsychopharmacol.* ***12***, 477–481; <https://doi.org/10.1016/s0924-977x(02)00056-1> (2002).
51. Smits, K. M. et al. Serotonin transporter polymorphisms and the occurrence of adverse events during treatment with selective serotonin reuptake inhibitors. *Int. Clin. Psychopharmacol*. ***22***, 137–143; <https://doi.org/10.1097/YIC.0b013e328014822a> (2007).
52. Smits, K. M. *et al.* The influence of 5-HTTLPR and STin2 polymorphisms in the serotonin transporter gene on treatment effect of selective serotonin reuptake inhibitors in depressive patients. *Psychiatr. Genet*. ***18***, 184–190; <https://doi.org/10.1097/YPG.0b013e3283050aca> (2008).
53. Quaak, M., van Schayck, C. P., Postma, D. S., Wagena, E. J., & van Schooten, F. J. Genetic variants in the serotonin transporter influence the efficacy of bupropion and nortriptyline in smoking cessation. *Addiction*. ***107***, 178–187; <https://doi.org/10.1111/j.1360-0443.2011.03534.x> (2012).
54. Dias, H., Muc, M., Padez, C., & Manco, L. Association of polymorphisms in 5-HTT (SLC6A4) and MAOA genes with measures of obesity in young adults of Portuguese origin. *Arch. Physiol. Biochem.* ***122***, 8–13; <https://doi.org/10.3109/13813455.2015.1111390> (2016).
55. Manco, L., Machado-Rodrigues, A. M., & Padez, C. Association study of common functional genetic polymorphisms in SLC6A4 (5-HTT) and MAOA genes with obesity in Portuguese children. *Arch. Physiol.* ***128****,* 1510-1515*;* <https://doi.org/10.1080/13813455.2020.1779312> (2022).
56. Gaysina, D., Zainullina, A., Gabdulhakov, R., & Khusnutdinova, E. The serotonin transporter gene: polymorphism and haplotype analysis in Russian suicide attempters. *Neuropsychobiology*, ***54***, 70–74. <https://doi.org/10.1159/000096041> (2006).
57. Toshchakova, V. A. *et al.* Association of polymorphisms of serotonin transporter (5HTTLPR) and 5-HTC receptor genes with criminal behavior in Russian criminal offenders. *Neuropsychobiol.* ***75***, 200–210; <https://doi.org/10.1159/000487484> (2017).
58. Ogilvie, A. D. *et al.* Polymorphism in serotonin transporter gene associated with susceptibility to major depression. *Lancet* ***347***, 731–733; <https://doi.org/10.1016/s0140-6736(96)90079-3> (1996).
59. Choi-Kwon, S. *et al.* Factors associated with post-stroke anger proneness in ischaemic stroke patients. *Eur. J. Neurol.* ***20***, 1305–1310; <https://doi.org/10.1111/ene.12199> (2013).
60. Baca-Garcia, E. *et al.* Association between obsessive-compulsive disorder and a variable number of tandem repeats polymorphism in intron 2 of the serotonin transporter gene. *Prog. Neuropsychopharmacol. Biol. Psychiatry*. ***31***, 416–420; <https://doi.org/10.1016/j.pnpbp.2006.10.016> (2007).
61. Florez, G. *et al.* Association between the Stin2 VNTR polymorphism of the serotonin transporter gene and treatment outcome in alcohol-dependent patients. *Alcohol Alcohol*. ***43***, 516–522; <https://doi.org/10.1093/alcalc/agn048> (2008).
62. Saiz, P. A. *et al.* Differential role of serotonergic polymorphisms in alcohol and heroin dependence. *Prog. Neuropsychopharmacol. Biol. Psychiatry*. ***33****,* 695-700; https://doi.org/10.1016/j.pnpbp.2009.03.016 (2009).
63. Saiz, P. A. *et al.* Interactions between functional serotonergic polymorphisms and demographic factors influence personality traits in healthy Spanish Caucasians. *Psychiatr. Genet*. ***20***, 171–178; <https://doi.org/10.1097/YPG.0b013e32833a20b9> (2010).
64. Sanjuan, J. *et al.* Mood changes after delivery: Role of the serotonin transporter gene.  *Br. J. Psychiatry.* ***193****,* 383-388; <https://doi.org/10.1192/bjp.bp.107.045427> (2008).
65. de Mel, S., Nordlind, K., Holst, M., Frohm-Nilsson, M., & Lonne-Rahm, S. B. Polymorphisms in the serotonin transporter gene of patients with atopic dermatitis-association with personality traits related to high level of anxiety. *Immunopharmacol. Immunotoxicol*. ***34***, 534–538; <https://doi.org/10.3109/08923973.2011.632636> (2012).
66. Kao, W. T., Chang, C. L., & Lung, F. W. 5-HTT mRNA level as a potential biomarker of treatment response in patients with major depression in a clinical trial. *J. Affect. Disord*. ***238***, 597–608; <https://doi.org/10.1016/j.jad.2018.06.035> (2018).
67. Jarrett, M. E. *et al.* Relationship of SERT polymorphisms to depressive and anxiety symptoms in irritable bowel syndrome. *Biol. Res. Nurs*. ***9***, 161–169; <https://doi.org/10.1177/1099800407307822> (2007).
68. Jasinska, A. J. *et al.* Amygdala response to smoking-cessation messages mediates the effects of serotonin transporter gene variation on quitting. *NeuroImage* ***60***, 766–773; <https://doi.org/10.1016/j.neuroimage.2011.12.064> (2012).
69. Mercer, K. B. *et al.* Acute and posttraumatic stress symptoms in a prospective gene x environment study of a university campus shooting. *Arch. Gen. Psychiatry*. ***69*,** 89–97; <https://doi.org/10.1001/archgenpsychiatry.2011.109> (2012).
70. Payer, D. E., Nurmi, E. L., Wilson, S. A., McCracken, J. T., & London, E. D. Effects of methamphetamine abuse and serotonin transporter gene variants on aggression and emotion-processing neurocircuitry. *Transl. Psychiatry*. ***2***, e80, 3; <https://doi.org/10.1038/tp.2011.73> (2012).
71. Shiroma, P. R., Drews, M. S., Geske, J. R., & Mrazek, D. A. SLC6A4 polymorphisms and age of onset in late-life depression on treatment outcomes with citalopram: A Sequenced Treatment Alternatives to Relieve Depression (STAR*D) report.  *Am. J. Geriatr. Psychiatry*. ***22***, 1140–1148; <https://doi.org/10.1016/j.jagp.2013.02.012> (2014).
72. Sulik, M. J. *et al.* Interactions between serotonin transporter gene haplotypes and quality of mothers' parenting predict the development of children's noncompliance. *Dev. Psychol.* ***48***, 740–754; <https://doi.org/10.1037/a0025938> (2012).
73. Taylor, Z. E. *et al.* Development of ego-resiliency: Relations to observed parenting and polymorphisms in the serotonin transporter gene during early childhood. *Soc. Dev*. ***23***, 433–450; <https://doi.org/10.1111/sode.12041> (2014).
74. Yohannes, A. M. *et al.* Serotonin transporter gene polymorphisms and depressive symptoms in patients with chronic obstructive pulmonary disease. *Expert Rev. Respir. Med*. ***15***, 681–687; <https://doi.org/10.1080/17476348.2021.1865159> (2021).
